# Hospital Sunday Special Supplement

**Published:** 1921-06-25

**Authors:** 


### Hospital Sunday, June 26, 1921.

### SPECIAL HOSPITAL SUNDAY FUND SUPPLEMENT.

### THE ACHIEVEMENT OF HOSPITAL SUNDAY.

During the past year the general public has become dimly aware that the finances of the voluntary hospitals were in a serious position, and our Hospital Sunday Number in 1921 almost corresponds with the recent report of Lord Cave's Commission on the hospital situation. The Report of this Commission stated that an emergency had arisen due to the state of prices after the war, and recommended a grant of one million to relieve it; but this grant would not have been put at so low a figure if existing sources of income, like that of the Hospital Sunday Fund, were not still the mainstay of our institutions. Those who are interested in Hospital Sunday will recall with pleasure the progress of the Fund during the past two years. In 1919 the grand total was £86,232, of which nearly half, as usual, was received from the collections of congregations. But last year the grand total rose to £121,058, an increase of £35,000, nearly one-half of which was again provided by the people in places of worship. It should encourage the supporters of the Fund to know that this magnificent improvement is not due to any fortunate accident, but seems to have been the reward of the energy and generosity displayed last year by every one interested.

With the same efforts the total should be maintained this year. Indeed, the same effort would probably increase it. Now Hospital Sunday stands precisely for the giving of personal service to any disinterested end, and in particular, of course, to the needs of the sick. The Hospital Sunday Fund was one of the earliest collective agencies for raising money for the voluntary hospitals, and nothing is more interesting than the way in which the method of the Hospital Sunday Fund has been employed or advocated for one section after another of hospital finance. The Hospital Saturday Fund is the complementary body, employing similar collections in workshops and factories; in fact, in the provinces the managers of the two Funds are often virtually one body. Nor has the method stopped there. For while it is true that we do not speak of Hospital Monday or Hospital Tuesday, it is now urged by those best qualified to judge that only by a weekly free-will contribution from wages and salaries in all places of business, offices, shops, as well as in the factories, can the income be regularly secured which the hospitals now require if they are to do all that is expected of them.

Twopence a week from every wage-earner in London would give to the London hospitals an assured income; and on Hospital Sunday we may well remember that such regular giving of small sums is the nearest expression of personal service that can be given in money. A small, regular weekly gift is a quiet, steady, invaluable assistance, similar to that personal service which is given freely by members of committees and other voluntary bodies by whom so much of hospital business is carried on. This being so, we hope that our readers will not be content with a gift to the Hospital Sunday Fund, but will further ask themselves if they cannot help to start weekly collections in their place of business. When we put forward this plan in our *Special Supplement* a year ago we determined to give our readers any encouragement that might be possible in 1921. A year later we are able to say that more than one London hospital has begun to encourage schemes of this character, and therefore that no more useful form of personal service can be given at this moment than the advocacy of regular collections in their places of business by those who hear the message of Hospital Sunday in church or chapel. We hope the clergy and ministers of religion in their sermons on Hospital Sunday will press this point home. If employers of labour will give their sympathy and support to a scheme of regular weekly collections in their factory or place of business it would be a direct incentive to employees to agree to support the great voluntary hospitals in this way.

In the present number we give an outline of the work of which the voluntary hospital is the centre, and of the needs of institutions in which the increase in income has failed to keep pace with the increase in prices and salaries. But among this mass of detail the reader must not lose sight of the principle for which the voluntary hospital stands. In days when everything is expected to be paid for at commercial rates, the hospital gives free treatment to the needy, is largely carried on by voluntary work, and is supported by freely-given subscriptions. Charges towards the maintenance of patients have been adopted to enable the institutions to continue during the present emergency, but this expedient does not alter their character or affect the value of their example. They remain expressions of human sympathy and generosity, whose purpose is to give and not to take, to help and not to profit, a very useful leaven in the competitive world of to-day. Thus support given at this moment is not merely to useful institutions in distress, but to the principle of personal service, a principle doubly in danger in the confusion which invariably follows a great war. It is natural, therefore, that the churches should have been quick to realise this situation, and to have made the splendid effort of last year, for not only have hospitals always been associated with religious institutions, but Hospital Sunday itself was the invention of a Church of England clergyman, the late Canon Miller, of Birmingham. It is extremely important that the great advance made last year should be preserved, for if the present difficulties were the result of four years of war, it is obvious that what it took four years to destroy cannot possibly be restored in an equal space, and not even three years have passed since the truce of November 1918. The achievement of Hospital Sunday last year is the more remarkable in that the number of congregations which took part in the collection was only twelve more than in 1919. The organisers this year can be relied on to repeat their efforts, but in the last resort the success of the movement depends on the individual givers; and it is to him and to her that we must leave this trust. All that we can record in these pages is but a summary of hospital activities. If the reader will grasp what principle inspires them, and how they relieve the homes of thousands in the dark hour of disease and suffering, he will feel that Hospital Sunday is in support of a great movement, is worth all that we can do to preserve it.

### WANTED A NATIONAL CAMPAIGN.

The apparent apathy with which the general public regards the present serious financial position of the great voluntary hospitals is surprising when we consider the important part which they play in the ordinary life of the nation. Has anyone not directly concerned with the health services ever paused to consider how the community would fare in the absence of the hospitals? It is not too much to say that to let them perish for lack of support would be to remove one of the props of civilisation. For the poorer classes, as well as for a large and increasing number of what is known as the middle-class element in the population, serious illness would take on a new terror. In countless cases it would not only mean the death of the sufferer, but it would dislocate the family life and rapidly turn a solvent home into a bankrupt home. When the poor wage-earner is beset with illness requiring careful nursing and skilled treatment, he is taken, almost as a matter of course, to the nearest hospital. When the struggling professional man, just able to afford the consulting specialist's fee, is told that an operation is necessary to save his life or to restore him to health and give him back the working capacity which is his chief economic asset, he knows that, if he cannot afford a first-class surgeon's heavy fee or the expenses of a nursing home, the doors of the hospital are open to him, and that within the hospital the skill freely expended on him is equal to that given to the richest man in the kingdom, able to command all the resources that wealth bestows.

### Casualties.

When the carter or the foot-passenger meets with an accident the ambulance takes him to the hospital. What would happen to the thousands of persons who meet with serious accidents in the street if there were no hospitals to receive them almost immediately? The out-patients' departments alone do a work on a scale so vast that the annual figures run into many hundreds of thousands. It is not our province here to discuss the research side of the activities of all the leading hospitals. But comparatively few people know even of its existence, and fewer still are aware of its direct bearing on the life of the nation. Without this research work, carried on almost in secret by a devoted band of clever men, the Ministry of Health and all the local agencies that seek under its guidance to prevent disease and to improve the amenities of life would be working almost in the dark, while the army of general practitioners who may be described as the watch-dogs of family health would be overwhelmed by the complexities and confusions of their task. There is not the slightest doubt that if the present policy officially laid down by the Ministry of Health is to be developed, or even maintained, on a national basis, the hospitals must not only survive, but must be greatly extended and placed in a position of unassailable security. They form an integral part of the friendly-society and panel system, and are linked up with every organised effort to cure disease and care for the sick poor. Their importance is increasing because the term "sick poor" has now a much wider significance than it possessed before the war, since a very large section of the middle classes which was formerly able to depend on its own resources in time of illness now has no margin of income to allow for these contingencies, and must rely more and more in the near future on the help which the hospitals can give. Hospitals should be regarded by every individual member of the community, no matter what his rank or status, as a form of national insurance for which, according to his means, he ought to make himself personally responsible. In the treatment of children and infants, and in the maternity departments especially, an immense development of hospital resources is required.

### Facts to Remember.

How many people realise, as a staunch advocate of the hospitals asked the other day, that every year 100,000 children die before birth, that upwards of 60,000 infants are dead before their first birthday occurs, and that more than 90,000 children have given up the struggle for life before they reach the age of five years? These are facts which should be urged in season and out from every public platform and every pulpit in the kingdom. The pivot of wealth has now been shifted largely to a new class of Englishmen not reared in the old tradition of a generous assistance of our most splendid charities. Ministers of religion and social organisations can do a great deal by combined and simultaneous effort to arouse among this new class the sense of its trusteeship in riches. But the obligation is one that cannot be confined to one class alone. It must be spread over the whole community. What we should like to see set going without a week's unnecessary delay is a great national campaign on behalf of the hospitals, the object of which should be not merely to lift them over temporary embarrassments, but to endow them permanently as a great British institution and a perpetual memorial of the public spirit and wise foresight of the nation.

### A DAY IN A HOSPITAL.

**By E. W. MORRIS, C.B.E., House Governor of the London Hospital.**

As I have been asked to write an article on the above subject, it is to be presumed that a day in a hospital is an experience which is novel to most of those who will read this supplement.

May I say that the fact that you do not know what it is to be in a hospital, and hundreds share with you this ignorance, is a very high testimonial to the hospitals. It is because, for some hundreds of years, they have been faithfully carrying out their duty that to-day you can boast you have never been in one.

And even if, through some sudden and unforeseen emergency, you can say that at some time you were a patient in a hospital, I do not think you can know much about the mass of machinery your admission set in motion. It was not visible from the bed on which you lay; great forces which were fighting on your behalf were, for the most part, out of sight; someone, who focussed the study and experience of years upon you while you were under an anæsthetic, did not wait until you had "come round" in order to be thanked; someone of great knowledge glanced down a microscope, and that glance searched out the cause of your trouble and determined a course of treatment which has resulted in your good health ever since, yes, and in other people's good health, too. But, as a patient, you knew nothing of the trained eye that, on your behalf, had looked down the microscope.

To the efficiency of what you did see—the care of the nurse, the skill and patience of the surgeon, the unconquerable hopefulness of the physician, the general organisation and method—you gladly pay tribute. But the half was not told you of what was being done for you, and, through you, for others outside.

A day in the life of the hospital! What day? We have a great choice of days. We could, if we wished, choose a day 800 years ago in the life of that fine old hospital, St. Bartholomew's. There it has stood in the Smoothfields—now Smithfield—just outside the City, since Henry I., on the death of his son in the White Ship, ceased to smile. It was founded by his jester, Rahere. There it has stood, unmoved and unmovable in its fixed intent of doing good while our beloved England fought and suffered, and fell and rose again under Norman and Plantagenet, Tudor, and Stuart. I would think it an honour to be a doorkeeper at St. Bartholomew's if thereby I might catch its fine unquenchable spirit.

St. Thomas's, too, can go back and back to the time when William Rufus lay dead in the New Forest and Walter Tyrrel fled the avenger. It has grown old in doing good, and yet shows no signs of age. Like Moses, its "eye is not dimmed nor its natural force abated."

What a choice of days we have! Come to more modern times, say 1800. My own hospital, "The London," had been humbly following the steps of its great exemplars for about sixty years. As hospitals go, we are an infant, born in 1740. We were born of poor but honest parents, and we were born in a public-house—namely, the Feathers Tavern, in Cheapside. Five men meeting there decided that with £100 which they had collected they would found a charity; which they did, The London Hospital. £100 would run "The London" to-day for four and a-half hours.

### One Hundred Years Ago.

Let us look inside. A bare, ugly building with whitewashed walls. The atmosphere is close and reeks with indescribable filth. The long barnlike wards are dreary and cheerless. They are lighted by candles. The beds are of all sorts and sizes and the bedding is grimy and foul. There are no nurses, but unpleasant women are hovering about called "watchers." They were usually aged widows, vicious and moralless, dirty and drunken harpies hired by the day, who saw their opportunity and used it—by stealing the very food of the sick; women who demanded tips before they would bring common necessities to the dying; women who could jeer at the unspeakable agonies endured in an age that knew not anæsthetics.

We see the patients lying in long rows of hopeless misery; for disease is not being fought, it is only being suffered, and it is perhaps a little better to suffer it in some sort of shelter than in the cold outside. Lounging about we see groups of noisy and common young men, the apprentices of the various surgeons. They are dirty, swaggering, and brutal. They bring their equally disreputable friends in "to see the show." And the show? If you listen you may hear a bell clanging. That announces the show. All the patients can hear it. That bell hangs over my head as I write. An inscription has been attached to it. It reads: "This operating bell was rung before the discovery of anæsthetics, to call the men to hold the patient down." The surgeon dressed in his oldest and filthiest clothes for his dreadful work. That is enough. We want to see no more of a day in a hospital a hundred years ago.

### A Modern Day.

Let us choose a modern day, a day in 1921, and, as I am to be your guide, we will choose the "London." Stand any morning at the gates in the Whitechapel Road. Watch the patients coming in, and watch them from the Porters' Lodge, where we shall not be noticed, because we must be careful not to hurt their feelings. You will see accident cases and emergencies of all degrees of seriousness coming in, or brought in, by the score. There is the strong man, but now very pale, and his mate helping him to walk, propping him awkwardly and making no fuss. That's a circular-saw case, I'll be bound, and his wrist tendons have been cut. It is not a life-and-death case, but it is life or death to that man and to his children that his arm should be saved.

A pair-horse van swings in at a trot. You saw nothing? No? "It" was lying on the floor of the cart.

**A Day in a Hospital—(*continued*).**

It came from the docks near by. That was something serious. You noticed the horses sweating? Here is a constable. He is carrying a child in his arms. He calls out something as he passes the gate. Yes, it is certain to be a "run-over." A taxi slips skilfully through; you catch sight of a white, hopeless face and someone else who is bringing "the case." Attempted suicide? Yes, that's what it was—we get a good many. A man and a woman are standing outside. They read every possible notice. They look up and down the hospital. Walk away a little, come back, and at last venture through the gate. He slips his arm through hers encouragingly, and leads her up the slope to the door of the hospital itself. I can tell you all about that. You need not ask them. She has been ill for months, getting slowly worse. He is hopeless, for she is very dear to him. At last he has persuaded her to come to "'orspital" and hear the worst. They are both in an agony of dread—each for the other. We are very used to those couples—not always young, sometimes, indeed, quite old—whitehaired and bent. They are the sweethearts. You will see lots of them in the course of the day. And here is a ragamuffin carrying a dog. He is slightly apologetic to the gate-porter. He saw "the blighter run over," so he brought it. He passes in.

The Receiving-room.

Now let us go into the receiving-room and see what happens to these people and the rest of the crowd that come through the gates in ceaseless procession. The receiving-room—the R.R. in hospital language—is the sieve of the hospital. There a staff of senior men, working in relays through the twenty-four hours, decide what is to happen to the patient. Three things may happen to him, depending on whether he is—shall we say?—little ill, or medium ill, or very ill.

If only a little ill, he is treated then and there; sometimes only A.O.—"advice only"—is needed. If "medium ill," more treatment may be required than can be given in the receiving-room, and the patient is given a pass to the out-patient department, where consultants and specialists sit daily and can give more careful attention to the case; if "very ill," the list showing where the empty beds are is consulted, the "man on full duty" sent for, and the case is admitted.

Watch these officers at work. Long experience in reading the signs has made them expert. The mother over-worried about her little baby who "looks anxious" is assured that nothing is much amiss and is comforted. A little nipper of eight brings up a littler nipper of two who has pneumonia, and is instantly "spotted." The small messenger is questioned. Mother was too busy to bring Joey up. She had got a job to-day charring, and father had been out of work for a long time. "Mother knew you was short of beds, but told me to say as how Joey was used to sleep with other people, and perhaps you could put him in a bed with somebody." We shall manage to solve the difficulty.

The suicide? Yes, she's being admitted, you see, and, by the number of bells that are clanging there seems to be need of hurry. The surgeon, who is "on full duty" and who lives, say, in Wimpole Street, has been told. He is on his way down, and he is hurrying. "Suicides" are always worth a good fight.

The Out-patient Department.

We must leave the receiving-room and go on. By a series of long corridors we reach the out-patient department. The central waiting-hall will hold two thousand people, and round this hall on three floors are the special departments. On a heavy day 1,500 to 1,700 out-patients may be treated. Everything that can be devised to destroy disease in man, woman, and child will be found in this out-patient department. There are suites of rooms for seeing ordinary surgical, accident, and medical cases; rooms for throat, ear, and nose diseases; rooms for eye diseases, fitted up perfectly for their work; the lupus-light department is there too; a room for radium treatment; rooms for radiography and for *x*-ray treatment; dental accommodation, splendidly equipped; baths for rheumatism; baths for skin diseases; costly apparatus for electrical treatment; departments for children; departments for women; a very fine fighting machine is this out-patient department.

In the basement are the pharmaceutical laboratories. Machines are stamping out tabloids by the hundredweight; cough-lozenges are being made by the ton. Trained men in the stores hard by are testing the specific gravity of ether—bought in 5-ton lots; the boiling-point of chloroform; the percentage of morphia in a recent delivery of opium from the docks. That is the best of being a hospital patient—you are in the hands of experts. An expert buys and tests the ether; an expert will administer it when the time comes; an expert will have sterilised all that is required at the operation—instruments and catgut and dressings; an expert will perform the operation; an expert will nurse you back to health and happiness.

I wish I could stay in this great out-patient department with you and talk to some of the patients. The superintendent could tell you a thing or two if you could get him to talk—which I never can. Every patient has come here via receiving-room, as you will have understood, and they all pass through the superintendent's hands here before seeing the doctor. His duty is to prevent abuse of the hospital's charity. "Do you judge by their clothes?" "Good heavens, no. See that woman sitting there, the third in the row, well she hired those clothes in order to come up to the hospital respectable. And that decently dressed young fellow, he is coming for *x*-ray treatment. Why, his clothes are his stock-in-trade, his uniform. He has little enough else—certainly he has not enough food. No, I don't go by their clothes."

Hullo! there's our sweetheart couple just leaving, on their way out. They have seen the doctor and are talking together. "What did I tell you, aren't you glad we came? He said it was nothing serious, and he'd have you right in a week or two." "Yes, that's what he said; and he told me to tell my man not to worry about me. So I'm telling you, Dick."

The Wards.

We must get on. We will leave the out-patient department and have a peep into one of the wards. There are sixty or so of them, so we cannot possibly see them all.

This is a Men's Surgical Ward, and is called "Wellington." It is visiting day, and at most of the beds are one or two relatives. The patients are

**A Day in a Hospital**—(*continued*).

from all parts. That man is a lighthouse-keeper from the "Wolf." This man is an old shepherd from the Sussex Downs, and the noise of London is the only complaint he has to make of hospital life. There is a docker, two or three of them; an old schoolmaster is in that bed; he has lived many years, and all of them lean ones; he has not many more to go, poor old chap. Two white-coated figures enter with a trolley. The theatres are at work. Wellington 15 is the next on the list in Theatre IV. He is sixth on the list of eight, and No. 5 is nearly finished. Wellington 15 is expecting those white-coated visitors. Had he been alone he would have dreaded them. But the Surgical Ward takes these things in its stride. He knows, too, that the King's own surgeon will deal with him, and deal with him exactly as if he were the King himself. He chatted with him yesterday, and the surgeon told him what he would have to do, and felt sure of an excellent result. Wellington 15 is lifted on to the wheeled couch and is pushed towards the door. No. 22, who went through the mill last week, and can now enjoy a pipe and has no respect for operations at all, calls out "Any flowers, Bill?" "No flowers, by request," says No. 15, and the door closes.

Theatre work never stops—visiting days or otherwise, weekday or Sunday, day or night—it never stops. In the war, during raid-time, it was exciting work. The theatres, with their great glass tops, are on the roof. My 'phone would report a raid as being imminent. But the surgeon must operate at once—patient is bleeding internally. How long has he? Telephone says ten minutes. He will go ahead then. A tarpaulin is over the glass roof and they will work in subdued light. He begins, and having begun, cannot stop. And the raid starts at the same time. Bombs drop heavily close round the hospital, and the glass windows rattle. Our own shrapnel clatters on the tarpaulin. The group in the theatre go quietly on —there is the surgeon, and the anæsthetist, the house surgeon, the sister, the nurse, two or three dressers, sterilisers—all standing quietly in white. There is no hurry. The surgeon turns to the anæsthetist and asks, "Is she standing it all right?"—just as he always does. "She is quite all right"—and they go on and finish.

But that *is* the voluntary spirit. It has taken some hundreds of years to make it bad form as well as bad work to neglect a patient. "Hospital first" is now taken for granted and influences everybody, from the boy who is peeling potatoes to-day for the patients' dinner, up to the Chairman, who can only be Chairman if he signed that very short agreement—"Hospital first."

Let us go up to the theatre floor. There are six theatres on this floor, although there are many others in different parts of the hospital. There is Wellington 15, just going in. He hands a postcard to Sister; will she please post it in the letter-box which stands in the corridor, post it while she is waiting—for the Sister of a ward always takes her patient to the theatre and waits throughout the operation. The postcard was to his mother and informed her that the operation was quite successful and he was getting on well—which turned out to be true, of course.

Here are the sterilising rooms, where experts are dealing with all that will be needed by the surgeons at the operation. On their conscientious work depends the life of the patient, as much as on the skill of the surgeon. They are handling instruments, and silk, catgut, and kangaroo tendon, bowls and dressings, and will see that all are "at the table" germ-free.

Now you will see what I mean when I said that those who never have entered a hospital benefit by the hospital's steady work. Every doctor in England was trained at one of them, and every nurse. The hospital's knowledge has become his knowledge. The hospital's victory over this and that foul disease becomes his victory, and for you. But far above all, he has had five precious years of opportunity of learning a very old lesson—that he is his brother's keeper and his brother is his: that to gain he must give; that to master he must serve.

### The Laboratories.

I must hurry you. I have only one day, and I could keep you for many. I want you to see the laboratories, where research is going on, where prevention of disease as well as its cure is being studied. This is the bacteriological laboratory, as you may see by its incubators and rows of microscopes. Here the full life of very small things is watched. What is their relation to human life? Are they harmful or beneficial? If harmful, what conditions foster their presence, what conditions will destroy them?

Here is the clinical laboratory, where complex investigations are being undertaken as to the chemistry of the body—the chemistry of the body in health and its chemistry when the small internal chemists are in mutiny and will not carry out their duties; this department will tell you how many red corpuscles there are in a cubic millimetre of your blood and how many white, and then will explain to you, if you wish it, what are the conclusions to be drawn.

I asked one of the clinical pathologists in charge the other day what was the motive that kept him and other laboratory workers like him at this work, so ridiculously underpaid. Why had he not gone into consulting practice in Harley Street? or even run a panel practice in the country, which would bring him in as many thousands as he was paid hundreds? He thought my question rather a foolish one, I think, although he replied courteously enough, "We fellows are not after money really. No merit in that: we are after something else. Some of us are born with an insatiable curiosity. We are always asking why? We are trying to find out causes, and every cause we can trace is a tremendous reward. We think it the very highest honour in the world to be associated, even indirectly, with a great man who has moved forward. Some of us like laboratory work because it is the best possible place from which to watch the general forward march of all ranks." "And what is your own motive?" I asked. "Well," he said, "when I was at Cambridge I saw three fine young fellows in the agony of tetanus, for which, then, nothing could be done. They all died.

**A Day in a Hospital**—(*continued*).

I said to myself, 'I'm going for that damned thing.' And here I am, still going for it, and others like it."

The X-Ray Departments.

And here is one of the $x$-ray departments; this one is for very expert diagnosis of all sorts of internal troubles, and a surgeon would not dream of operating without a report first from this department.

There are other $x$-ray departments, not for taking radiographs at all, but for giving huge doses ("massive doses" they call it) of $x$-rays, which are filtered through aluminium plates of varying thickness before reaching the patient; by this means the dangerous rays are filtered out, the beneficent ones pass. I should like to have shown you some cases of inoperable cancer after treatment of these "massive doses" and I feel, from what I am told, that the hospitals are on the verge of most important discoveries in controlling this curse. The best brains in them all are working at it.

I should like to take you to so many other places, but I should tire you out; the laundry, a charity within a charity, where ninety girls are employed and which washes 60,000 pieces a week; the kitchens which cook for some 2,000 people a day, and have to measure punctuality in quarter minutes; the dispensary; the Tyrnauer baths; the Swedish exercise department, where bent-back crippled children are being made straight and take a most astounding interest and pride in the process.

The Children.

On our way out I will just let you peep into a children's ward. Here is one—"children's, surgical"—full of "run-overs," scalds, burns, "falls out of window" and cases of that kind.

The gentleman sitting at the end of the ward by a cot is one of our best-known surgeons, who happens to be passionately fond of children. He wastes a lot of time romping with these children. He is engaged, I see, in the very important work of mending the child's doll. He mended the child herself last week.

The Hospitals' Great Crisis.

And now the hospitals are fallen on evil days, and some of them, including my own, have had to face the awful tragedy of shutting up some of the wards. More of them are about to do so. To appreciate this you must visit the receiving-room and note the anxiety of the officer who is forced to send away a case he knows he ought to admit, and would admit but for those dreary empty beds upstairs. And it is not the hospital's fault. The crash has come for two reasons: first, that the treatment of disease, owing to modern discoveries, has become increasingly costly; and secondly, of course, the war.

At the beginning of this paper I told you of the hospital as it used to be. It was a place where suffering and disease were just endured. It was in no one's conception that disease ought not to be endured at all, but fought and resented and destroyed, everywhere and everywhen and at sight. And so the giant, like Goliath of Gath before the army of Saul, stalked up and down our land—plague, and smallpox, and consumption—and laughed our hospitals to scorn.

But there is always a David. Pasteur was a David; so was Lister, and Jenner, and Ross, and those great Americans, Reed, Carrol, and Lazear.

The giants are no longer scoffing at us, thanks to these Davids. They dread us. But they have one hope—that the cost of the armament and ammunition will be prohibitive. Their hope is our horror and nightmare.

Empty Beds.

You ask me why we cannot still kill Goliaths with pebbles from the brook, as of old. Well, please remember that only one giant was ever slain so cheaply. The study of disease is a costly thing. I have seen the "cost per bed per annum" creep up from £80 to £200, and more. How was that extra money spent, and was it well spent? You ask, naturally enough perhaps, whether more patients were passed through the beds at the higher cost. Yes, certainly, twice as many. But that is not the true test or measure of our success at giant-slaying. Then you ask me whether the death-rate fell. Yes, it has. But that is not the true test either. The real test is the number of persons who will never need to enter a hospital at all because of the work of the hospitals. That is the standard by which to measure the strength of your fighting power; not the full beds, but the empty ones are what we ought to be proud of. When our fever hospitals lie empty and our sanatoria are deserted, and our smallpox hospitals are shown as a curiosity, a memorial of a scourge that is dead, then we shall know that we have fought a good fight.

These old hospitals of ours have honestly done their best for hundreds of years, and now, when victory over disease seems almost in sight, a victory past all their first imaginings, some may have to close their doors. When that happens there will be merriment in the army of the Philistines; Goliath will go on bragging, and every harlot in Gath will deck herself in scarlet.

You say that it is useless for the hospitals to progress unless there is general progress. That to cure the little child of a tuberculous hip is useless when the child will only return to an insanitary home and relapse. We also have said that many times. But it is better to go on. You say that it were better that the poor woman died than recover and pass into a life of shame. We, too, have wondered at the "great enigma of permitted wrong." Nevertheless, it is better to go on.

You may think of many reasons, if you wish to, for not helping the hospitals at this most critical period of their existence. May I give you a reason why you should? I quote from a very old Book:

"Is not this the fast that I the Lord have chosen? To undo the heavy burdens and to let the oppressed go free, and that ye break every yoke? Is it not to deal thy bread to the hungry, and that thou bring the poor that are cast out to thy house?"

### WHAT THE LONDON HOSPITALS COST.

### Three Million Pounds Needed for Two Million Sufferers.

Early this year the Minister of Health, in consequence of the solicitation of many people in and out of Parliament, appointed a Committee "to consider the present financial position of the voluntary hospitals." The position was one which urgently called for such an inquiry. The Health Ministry was faced by a possibility of a service costing annually eight millions of money either becoming a charge on the State or reaching a stage when its acknowledged efficiency would suffer for need of adequate financial support. For clearly excellent reasons the gentlemen serving on this Committee were purposely chosen from those who had no immediate connection with hospitals, thus it was made possible to institute an inquiry completely unprejudiced and unbiased. As a result, after a few busy months, this Committee, under the presidency of Viscount Cave, issued a report, and were able to say that "the voluntary hospital system, which is peculiar to the English-speaking peoples, is part of the heritage of our generation; and it would be lamentable if, by our apathy or folly, it is allowed to fall into ruin." The chief note of the report is that the system is sound at the core, but is passing through a crisis which it is possible to relieve by a very moderate amount of help from public funds, and that, given also the whole-hearted help of the public, the hospitals will emerge from their troubles greater than ever.

There is a general but erroneous idea that public support of hospitals has fallen off. This is wrong, on the contrary that part of the income which comes from voluntary contributions has considerably increased recently, and has doubled during the past ten years. This is clearly demonstrated by the table on this page showing the income of Metropolitan hospitals and dispensaries for ten years, including 1919, the last year for which exact figures are available. This fact is confirmed by the rather less full statistics for 1920 included in the Cave Report. The point is that increased though these figures are, they have not kept pace with growing expenditure. Hence the hospitals of London are at the present time spending £400,000 a year more than they are receiving. Little wonder, therefore, that great hospitals like the London and King's College have severely to curtail the relief they have hitherto afforded to necessitous sick persons.

**METROPOLITAN HOSPITALS' AND DISPENSARIES'**

**Income in the ten years 1910 to 1919.**

| Year | From the Living | | From the Dead | | | | Total Income |
|---|---|---|---|---|---|---|---|
| | Subscriptions, Donations, Patients' Payments, etc | Percentage of Total | Investments | Percentage of Total | Legacies | Percentage of Total | |
| | £ | | £ | | £ | | £ |
| 1910 | 703,841 | 50 | 309,320 | 22 | 394,478 | 28 | 1,407,639 |
| 1911 | 738,622 | 53 | 329,253 | 24 | 322,949 | 23 | 1,390,824 |
| 1912 | 741,439 | 55 | 338,425 | 25 | 272,090 | 20 | 1,351,954 |
| 1913 | 845,908 | 52 | 349,295 | 22 | 424,339 | 26 | 1,619,542 |
| 1914 | 801,239 | 49 | 366,841 | 23 | 451,569 | 28 | 1,619,649 |
| 1915 | 978,294 | 62 | 368,536 | 23 | 242,049 | 15 | 1,588.879 |
| 1916 | 1,143,417 | 63 | 382,377 | 21 | 301,282 | 16 | 1,827.076 |
| 1917 | 1,300,539 | 65 | 414,636 | 21 | 290,025 | 14 | 2,005,200 |
| 1918 | 1,602,717 | 68 | 430,566 | 18 | 343,274 | 14 | 2,376,557 |
| 1919 | 1,522,342 | 69 | 450,858 | 20 | 236,072 | 11 | 2,209,272 |

Turning to the figures again we notice the remarkable fact that living people give not much more than twice what has been provided by benefactors who are no longer with us, or in other words, one-third of the burden has been lifted from the shoulders of the present generation. But the fact that the hospitals have already a nest-egg should be an incentive to further giving to provide what, after all, in a city of the magnitude of London, is a comparatively small sum.

To secure efficiency, to keep what hospital accommodation exists going, to provide moderate but natural extension, a sum of three millions a year is needed. Four-fifths of this may be said to be secure, and we appeal for the remainder. And what could plead more strongly, more eloquently, than the statistics to be found on page 13? To treat nearly two million patients, each of them remaining in a ward of a hospital for several weeks or attending a number of times in the out-patient department we need three million pounds, or an average of thirty shillings for each patient. The economic value of the transaction is self-evident.

To take one class of patients — those dealt with at the eye hospitals or the ophthalmic departments of general hospitals. Apart from the dictates of humanity, to arrest or to cure eye trouble in an individual is to give back to the community a citizen who has twice or more than twice the economic value of one who would become blind or acquire permanently defective eyesight. Another class which also makes a strong appeal is that represented by the maternity cases and those of diseases of women. The woman, the mother, is the very key of the home life of the country. That she and her children should have the best attention that medical and surgical skill can give should be the first and most earnest consideration of everybody.

It can be confidently claimed this Hospital Sunday that our hospitals labour at the primary factors of economic wellbeing, and that health being wealth they are necessary to the prosperity of our country. To all, then, should they appeal—to those who require hard facts unmixed with sentiment to loosen their purse-springs as well as to the many who need no further consideration than that an opportunity arises to extend a helping hand to suffering humanity at an especially anxious time.

### THE NATION'S NURSES.

Among the benefits conferred by the voluntary hospitals upon the public that of maintaining the nursing service of the country is one of the greatest, and is certainly the least clearly understood. Modern surgery depends largely for its phenomenal success on a fine co-operation between the medical and the nursing professions. At every turn the surgeon is supported by the attendant nurses. He can depend on them for the preparation of the patient for the operation and for the aseptic condition of his instruments, his overall, his dressings, his surroundings. If any link in this chain were to give way disaster would ensue. He knows none will give way. At every moment during the operation nurses are his prescient assistants, anticipating his wants, instantaneous in obedience, vigilant, untiring, responsive to the slightest gesture. The moment the operation is at an end the surgeon hands over his patient to the nurses without the slightest misgiving. Another operation may await him; or, his day's work finished, he may empty his mind of its preoccupations. His concern with the patient is over until the next day.

Without skilled nurses the surgeon would be compelled to superintend personally every stage in the recovery of his patient from the anæsthetic and subsequent treatment. He could get through but half the work he is now able to undertake. His fees would necessarily be double. Moreover, there is a wide range of offices performed by skilled nurses for the comfort and speedy recovery of their patients, which lies completely outside the very different knowledge and skill of the surgeon. The training of the medical student does not necessarily embrace any knowledge at all of nursing. For all the dexterous services which contribute so largely to the well-being, and indeed the safety, of his patient the surgeon must rely, and does so without apprehension, on the nurses.

The modern nurse training-school attached to a voluntary hospital holds as its highest ideal the training of women to perform with meticulous care the critical duties of a surgical nurse. Poor-law infirmaries, with a few notable exceptions, lack the material for giving this training. It is the glory of the voluntary hospitals that they anticipated the need, invented the procedure, and perfected the mode of training surgical nurses. It has not been done without long and sustained effort, continual experiment, and great cost.

The nurse training-schools depend for success on their ability to attract probationers capable of responding to the carefully planned course which is necessary for the evolution of the first-rate surgical nurse. To this end a vast collegiate establishment has grown up in connection with all the major hospitals. Probationers must reach a certain standard of education. They must be provided with cheerful and healthy quarters. They must be under continual theoretical and practical instruction. Their work is exacting, and without recreation their health fails. The influence surrounding them must be good. Their nursing teachers and superior officers must be women of education, imbued with sound knowledge of their profession and inspired by a true vocation. The training aimed at is no mere cramming with information, no mere mechanical precision of the fingers. It is a radical cultivation of the faculties. Eye, ear, touch, but most of all the heart, are to be trained in unswerving response to a high ideal of personal service before the nurse whom the country needs is ready for duty.

Independent of salary, which may fairly be reckoned as payment for services in the wards, the training of each nurse cannot be estimated to cost the hospital less from first to last than £250. No grant has ever yet been made to the hospitals for the educational services they render. Their colleges have given a lead in educational methods which has been hitherto little observed. They may justly boast that in the development of the sterling qualities which go to make up what is called "character" their methods have never been excelled. They have a great claim on the public generosity for the production of the nurses who form a vital link in the national medical and surgical service. They ought undoubtedly to receive adequate subsidies from the State to ensure the continuance of their educational work at its present high level.

### St. Thomas's Hospital.

Hospital Sunday corresponds very closely this year with the anniversary day of St. Thomas's Hospital. Fifty years ago Queen Victoria, on June 21, 1871, opened the new buildings on the Embankment, the foundation-stone of which had been laid by her on May 13, 1868. The hospital was built at the same time as the Albert Embankment, a great part of which was made on flats recovered from the river. The Bishop's Walk ran between the Archbishop's Park and many old shops and boat-houses that stood on the banks of the river. To this new site was moved the ancient hospital which stood of old in St. Thomas's Street, Southwark. First endowed by Peter de Rupibus in 1213, the hospital was re-endowed and granted a Royal Charter by Edward VI. in 1553, and within the Close of the old building Guy built his hospital in 1724. Before the war, the assured income of St. Thomas's Hospital was practically sufficient to meet all expenses. But high prices and heavy rises in wages have very seriously affected this ancient charity. The total of ordinary expenditure in 1920 was £171,983, a very serious increase on the £76,174 which was spent in the year 1913. Some idea of the increased demands on the resources of the hospital are given by the following items :—

| | 1913. | 1920. |
|---|---|---|
| Provisions ... ... | £14,415 | £32,133 |
| Surgery and Dispensary ... | 7,480 | 18,344 |
| Salaries and Wages ... | 28,618 | 66,034 |
| Rates and Taxes ... ... | 3,117 | 5,960 |

The income from invested property in 1920 was £65,591, compared with £64,173 in 1913. To meet the increased cost, King Edward's Fund made a grant in 1920 of £9,000, and the Hospital Sunday Fund a grant of £3,600. From various payments, including Government grants for venereal disease, the total amount received from "patients' payments" was £27,505 last year.

### A Debt and an Overdraft.

There is an accumulated debt due to the bankers of £83,000, with an overdraft of £16,000. Funds are, therefore, very urgently needed at this hospital to enable the Governors to carry on the work and to take steps for opening the three closed wards by which poor patients are debarred from the advantages of some of the best hospital treatment which can be given in London. Patients are recognising the importance of maintaining the voluntary principle and are contributing to the utmost of their means. About £300 per week is being received at the present time from this source alone, but all who can afford to do so are invited to become regular subscribers, for it must not be forgotten that it is in our great voluntary hospitals that our medical men are educated and nurses trained for service to all who may need their help in their private homes. 8,362 in-patients were treated in 1920; 69,658 out-patients made 379,329 attendances. There are 632 beds for in-patients, but, of these, ninety beds are still closed for lack of means.

### THE SOCIAL SERVICE SIDE OF A HOSPITAL'S WORK.

### The Almoner's Department.

"How many desolate creatures on the earth
Have learned the simple dues of fellowship
And social comfort in a hospital."
*E. B. Browning.*

THE modern hospital is indeed an amazing and cheering institution. The most scientific methods, the greatest skill and learning are at the service of the patients. The wards are flooded with the selfless dèvotion and loving care of the sisters; the best is never too good for the patients, many of whom, for the first time in their lives, realise what sympathy, peace,. and social comfort really mean. Not merely is the treatment up-to-date and efficient, but in many hospitals the patient is treated as a whole person, with the understanding that illness is no isolated phenomena, but something which can only be treated successfully in its relation to life. Through the gates of a hospital flows a never-ceasing, ever-changing stream of sick and anxious humanity, to whom ill-health has come, if not as a calamity, at any rate as a desolation of life. Some of these are well able to manage their own affairs, but a large number owe their illness to ignorance, to folly, to a lack of means, and it is to help these that the almoner's, or social service department, exists.

THE HELPING HAND, HEART, AND HEAD.

The doctor advises the ideal course of treatment, but to many it is little more than a mockery, if it is not followed up by further advice and help to bring it into the realm of practical politics. How is the mother of a large family to leave her children and come into a hospital for a most necessary operation? How is she, without help, to have rest and change of air afterwards if her husband's income is quite insufficient for even the ordinary everyday expenses? How is the single girl, sick with shame and anxiety, to do the best for her unborn baby if there is no one to stretch out a hand of fellowship and give practical, knowledgable advice and help? In such cases, unless help is forthcoming, the words of wisdom of the doctor remain, for the most part, words. One can well understand an American doctor's remark that before he had a social service department he sometimes felt like the Mad Hatter in "Alice in Wonderland"—

"'Have some wine,' said the Hatter.
'I don't see any wine,' said Alice.
'There isn't any,' said the Hatter."

THE LINK WITH OUTSIDE ASSOCIATIONS.

It is the almoner's duty to represent the patient's home life in the hospital. A doctor wants to know where a certain patient comes from, what are his surroundings, can his wife look after him? The almoner's task is to regard each out-patient as an in-patient living at home. It is obvious she cannot deal with the overwhelming numbers alone. In some few cases the confidential nature of the information she may have necessitates independent action on her part, but as a rule she acts as a link between the hospital and the various philanthropic agencies outside who exist to help the sick and distressed, and whose existence is generally unknown to the people who most need them. Instruments, convalescence, extra nourishment, loans, and monetary aid are frequent needs, and to every known source of help the patient is guided. The clergy, the school authorities, the employers are constantly consulted, and a most valuable source of help to patients is the Hospital Sunday Fund.

The almoner's work is not bound by any definite and material limits. It is her duty to study everyone who is sent to her in need, Jew or Gentile, educated or uneducated, and the ways of help are as varied as the patients. But there is one object always in view—that everyone coming to the hospital shall gain every possible advantage from treatment and that they shall not lose the good which the hospital is bestowing through poverty, ignorance, or lack of a helping hand.

### One Year's Roll-Call of the Sick.

**Patients Treated at Metropolitan Voluntary Hospitals and Dispensaries and M.A.B. Infectious Hospitals.**

| | |
|---|---|
| Sufferers needing Surgical Aid ... ... ... | 579,904 |
| Sufferers needing Medical Care ... ... ... | 645,838 |
| Sufferers from Eye Troubles ... ... ... | 108,071 |
| Diseases of Women (including Lying-in) ... ... | 201,427 |
| Diseases of the Ear, Nose, and Throat ... ... | 52,837 |
| Sufferers from Skin Diseases ... ... ... | 41,296 |
| Consumptives ... ... ... ... ... ... ... | 65,399 |
| Fever Patients ... ... ... ... ... ... | 21,287 |
| Paralysis and Epilepsy ... ... ... ... | 21,027 |
| **Total ... ... ...** | **1,737,086** |

The above figures include only the in-patient cases treated to a termination in the wards of the hospitals and the number of new out-patient cases treated in the out-patient departments and dispensaries; 173,013 of these patients were tended in hospitals for children. They claim our sympathy and help. To the vigorous, to those in health who are able to provide for their dependants, to those who know what ill-health means, who have suffered from disease of one kind or another, and who, either in the hospital or under the skill and care of the doctors and nurses trained in the hospitals, have been restored to health and usefulness, we confidently appeal on behalf of the London hospitals.

THE following Scottish hospitals and institutions benefit by shares in the residue of the estate of Mr. W. M. Ledingham, of Aberdeen: Aberdeen Royal Infirmary and Royal Hospital for Sick Children, Waringfield Hospital, the Ophthalmic Institute, Aberdeen Dispensary, Maternity Hospital, and Nursing Association

### HOSPITALS AND THEIR SPECIAL NEEDS.

**BECKENHAM COTTAGE HOSPITAL.**—This institution was founded nearly fifty years ago, and provides twenty-five beds for a population of over 50,000. It is mainly supported by contributions by or on behalf of patients and by subscriptions and donations from the public. The hospital affords, when circumstances permit, accommodation for four patients paying the full cost of maintenance.

**BRITISH HOSPITAL FOR MOTHERS AND BABIES (National Training School for District Midwives), WOOD STREET, WOOLWICH.**—The new hospital is already half-built; it is to hold forty maternity patients instead of fourteen as at present, and will provide a training of one year's duration, specially adapted to the needs of district midwives. Building operations will have to be suspended shortly unless more funds are forthcoming. £50,000 need to complete the work.

**CHELSEA HOSPITAL FOR WOMEN** makes an urgent appeal on behalf of those on its "waiting-list," whose admission is greatly delayed for want of accommodation. As soon as it can raise £14,000 and complete with £15,000 in hand the provision for a nurses' home, the remaining half of the hospital wards will be freed for patients. It is calculated that, pending that provision, 700 patients annually are spending months of pain which they might otherwise be saved.

**CHILDREN'S HOSPITAL FOR THE TREATMENT OF HIP DISEASE, SEVENOAKS.**—Maintains forty beds and receives patients from all parts of the country, and keeps them for any length of time required to effect a complete cure, which cannot be done in a general hospital. Annual subscriptions and donations are greatly needed.

**CITY OF LONDON HOSPITAL FOR DISEASES OF THE CHEST, VICTORIA PARK, E. 2.**—It was only by means of special grants from King Edward's Hospital Fund and the National Relief Fund that the hospital escaped a large deficiency last year. It can hardly be hoped to reduce the expenditure below £32,000 in the present year. In addition to sanitary and other structural improvements urgently required, the Committee have to face expenditure for the maintenance of the sanatorium of twenty beds at Saunderton, Bucks, for which further income of £1,000 is required for the present year.

**CITY OF LONDON MATERNITY HOSPITAL, CITY ROAD, E.C. 1.**—Since its establishment in Aldersgate Street in 1750, the hospital has delivered over 81,000 in-patients. Last year the admissions numbered 1,916, of whom 416 were free. The hospital caters for the poorest, many of whom are unable to afford even the smallest donation. There is a Consultative Maternity Centre, and twice weekly the Ante-Natal and Child Welfare Centres are open, the attendances at which last year totalled 10,839. Pupil-midwives and nurses are trained, and medical students admitted to the practice of the hospital. Its great need is a convalescent home, which would enable the admissions to the hospital to be doubled. The bank debt now stands at £4,250.

**CONVALESCENT HOME FOR POOR CHILDREN, ST. LEONARDS-ON-SEA.**—The above Home, situated in a beautiful position overlooking the sea, carries on splendid work year by year in restoring to health children from the poorest parts of London and elsewhere. There are seventy-five beds, and last year 520 children were admitted. In common with most institutions of this class, the Committee are urgently in need of funds, and are most anxious to obtain new subscribers, who receive a letter of admission entitling a child to a three- or four-weeks' stay at the Home for every £1 11s. 6d. subscribed.

**DREADNOUGHT SEAMEN'S HOSPITAL, GREENWICH.**—For some years past the Seamen's Hospital Society has been fortunate enough to meet its expenditure, thanks to the generosity of those who realise what is due to the men of the sea. In 1920, however, although every effort was made to obtain the necessary funds and to keep expenditure as low as possible, the balance showed a deficit. Since the Society was founded in 1821 (100 years ago), the annual number of patients treated has grown from about 500 to over 18,000, and the Society now maintains three hospitals, a convalescent home, a sanatorium for consumptive sailors, and a dispensary. A few years ago our very existence depended upon the sailor, and at the present time our commercial activities rely to a great extent upon his labours.

**EAST LONDON HOSPITAL FOR CHILDREN** ended the year 1920 with a deficit of over £11,000. The increased cost, the rise in wages, and a very large bill for repairs which were absolutely necessary for the maintenance of the building are responsible for this large deficit. The ordinary income has always been inadequate, and never more so than at the present time. Funds are sorely needed to clear off the debt, which hampers the Committee at every turn.

**EVELINA HOSPITAL FOR CHILDREN, SOUTHWARK BRIDGE ROAD, S.E.**—The "Evelina" is the largest children's hospital to serve the extensive area of South London; further, it is situated in a most destitute district, and suffers accordingly, as it is rarely seen by the well-to-do. It is imperative to obtain *immediately* substantial donations and many new subscribers. Although this hospital received a liberal grant from the National Relief Fund, 1920 closed with a very considerable deficit (over £3,000), notwithstanding that every care and economy is being exercised by the Committee of Management and the officials. The Committee are anxious to raise £10,000 additional to the ordinary income to extinguish the deficit and to provide the amount required for entirely repainting the hospital.

**FLORENCE NIGHTINGALE HOSPITAL FOR GENTLEWOMEN, 19 LISSON GROVE, N.W.,** is maintained for the benefit of ladies of small means, such as governesses, hospital nurses, artists, musicians, etc., and the relatives of clergy, naval, military, and professional men. It contains thirty-eight beds, and 460 patients were admitted in 1920. Through the generosity of physicians and surgeons, the patients receive free medical and surgical treatment, but as the fees, which range from 30s. to 63s. per week, do not cover the cost of maintenance and nursing, the balance must be made up by voluntary contributions. It is estimated that there will be a deficit of £2,000 at the end of the year unless help is forthcoming.

**GERMAN HOSPITAL, DALSTON.**—Receives all sick poor, irrespective of creed or nationality. Fifty per cent. of both in- and out-patients are British born. Last year 1,807 in-patients and 11,606 new out-patients were treated. Deficit on December 31 last, £4,380. One ward of eighteen beds closed for want of funds. New out-patients' department, dispensary, and mortuary buildings badly needed. Increased and new support urgently required to meet the present exceptional cost of upkeep.

**GREAT NORTHERN CENTRAL HOSPITAL, HOLLOWAY ROAD, N. 7.**—The hospital is the only general hospital in North London, and with its branches has accommodation for 310 beds—namely, 220 at its headquarters in the Holloway Road, for the most urgent cases, sixty in the Great Northern Hospital of Recovery at Southgate, to assist in quick restoration of health (a method of treatment recommended in the Lord Cave Committee's Report on Voluntary Hospitals), and thirty in the Reckitt Convalescent Home at Clacton-on-Sea. Patients contribute towards cost of treatment in the Great Northern and its branches as far as they are able, the public being asked only to make up the difference between these payments and the actual cost of maintentance. At present the hospital owes its bankers £14,000, and a further sum of £15,000 is needed to pay off the present debt.

**GROSVENOR HOSPITAL FOR WOMEN, VINCENT SQUARE, WESTMINSTER, S.W. 1,** is in urgent need of money to meet a large overdraft at the bank, and for funds to carry on. Much difficulty is experienced in obtaining new subscribers to the hospital, which is small and therefore not well known, and the patients treated are from no particular locality. The financial position is the cause of great anxiety.

**GUY'S HOSPITAL, LONDON BRIDGE, S.E. 1.**—The immediate position which the Governors have to face for the year 1921 is the necessity of finding a capital sum of £75,000 to pay for the completion of the extension to the nurses' home and of the new massage department and the urgent enlargement of the central power-station. Moneys will also have to be found to defray the deficit of £21,000 on last year's working of the hospital, and to provide the necessary funds for maintenance during this year at a cost which will certainly remain much in advance of recent years. The Governors would therefore once more earnestly appeal for further support in the shape of donations and legacies, and express the hope that in the near future some generous donor may arise who may be moved to rival in his generation the noble endowments that Thomas Guy and William Hunt made in the cause of charity in the years 1724 and 1829.

**HAMPSTEAD GENERAL AND NORTH-WEST LONDON HOSPITAL, HAVERSTOCK HILL, N.W. 3.**—The Hospital serves the North-West London district, including the populous area of Camden and Kentish Town. The number of patients daily under treatment in the wards at Haverstock Hill averages 112, and many out-patients are dealt with in the well-equipped department situated at Camden Town. It is estimated that a sum approximating £26,000 will be required to meet the total expenditure incurred during the current year.

**HOME HOSPITAL FOR WOMEN, 187 HIGH STREET, STOKE NEWINGTON, N. 16.**—There is accommodation for twenty-five patients, and in addition to taking the ordinary cases, the institution meets the needs of those who require a certain amount of surgical and medical care, but are not ill enough for admission to the general hospitals; maternity cases are also taken. This hospital is greatly in need of funds for building nurses' quarters in the garden.

**HOSPITAL FOR CONSUMPTION AND DISEASES OF THE CHEST, BROMPTON, and SANATORIUM AT FRIMLEY** contain nearly 500 beds and admit for treatment over 2,000 in-patients yearly. There are also over 40,000 attendances in the out-patient department. The hospital represents a complete and up-to-date unit for dealing with consumption and other diseases of the chest. Its equipment includes special departments for *x*-ray, dental, cardiograph, throat, nose, and ear, clinical and research laboratories, and a staff of lady almoners to assist the patient, arrange as to convalescence, home conditions, after-care, and in many other ways. In addition every patient upon being passed as suitable is transferred to complete the necessary treatment at the sanatorium at Frimley.

**HOSPITAL FOR EPILEPSY AND PARALYSIS, MAIDA VALE, W.**—The hospital's greatest need is an extension of its out-patient department, which is truly an out-door department in that a great many patients have to wait outside on account of the great pressure for space. The estimated cost of this valued improvement is £8,000, towards which £4,500 has been received. The hospital requires £12,500 for its annual maintenance, and gifts would be gratefully acknowledged by the Secretary, Mr. H. W. Burleigh.

**HOSPITAL FOR SICK CHILDREN, GREAT ORMOND STREET, W.C. 1.**—The fact that the committee owe the hospital bankers at the present moment £20,000 and they have had to raise another £10,000 on mortgage of a reversionary legacy, shows that the financial position of the hospital is becoming desperate, and unless some material assistance is immediately forthcoming, part, or the whole, of the hospital will have to be closed. H.R.H. the Prince of Wales, President. Acting Secretary, Mr. James McKay.

**KING'S COLLEGE HOSPITAL, DENMARK HILL, S.E.**—This is the most modern and best-equipped hospital either in or out of London. There is accommodation for 400 in-patients and considerably over 49,000 casualty and out-patients receive attention each year with 206,000 attendances. The cost of carrying on the work of this great institution is not less than £130,000 per annum, and the income from all sources is not more than £57,000 a year, leaving an additional £73,000 to be raised. The total debt at December 31, 1920, amounted to £100,000. By keeping in use the 400 beds this debt has been added to at the rate of at least £1,000 per week. With the greatest reluctance, and in order to avoid a worse disaster, the committee of management have been compelled to close 160 beds spread over seven wards. The closing of the 160 beds at such a time as the present is lamentable yet unavoidable, so far as the committee of management is concerned, as they could not possibly continue adding to the debt of £100,000 at the rate of over £1,000 per week. An earnest appeal is made for new and increased annual subscriptions and contributions.

**LONDON FEVER HOSPITAL.**—The above hospital is not rate-supported, and was founded in 1802 by the inhabitants of London for the prevention and cure of infectious fevers. It is, therefore, the parent hospital in London for infectious diseases, and is now in danger of having to close its doors for want of funds. Over 100,000 patients have been treated since 1802, and nearly 1,000 yearly for the past three years. There are 178 beds, and patients pay an amount towards their cost, but the expenditure absolutely necessary for efficient work far exceeds the receipts, and the committee have been compelled to borrow money from their bankers, and there is now a very considerable overdraft.

**LONDON HOMŒOPATHIC HOSPITAL, GREAT ORMOND STREET. W.C.1.**—There could be no more eloquent testimony to the beneficence and extent of the work accomplished at the London Homœopathic Hospital, Great Ormond Street, W.C. 1, than that furnished by the enormous growth in the numbers of in- and out-patients treated in the last twenty-five years. Of the former, 31,713 have been admitted, and 347,623 out-patients, with a total number of 1,208,804 attendances, have received treatment during this period. The need for further development is strikingly apparent, but lack of funds bars the way.

**LONDON HOSPITAL, WHITECHAPEL, E.**—One word —" Desperate "—sums up the present position of England's biggest medical charity—the London Hospital. It has, indeed, actually closed 200 beds and cut down the out-patients by half owing to lack of funds. The financial position stands thus : with a certain income of £36,000 a year, the hospital has to meet essential expenditure amounting to over £250,000 a year. It owes more than £50,000. Every security is pledged. It is the blackest hour in the whole 180 years of the hospital's history.

**MARY YOLLAND INTERMEDIATE HOME FOR INCURABLE GIRLS, UPPER HALE, FARNHAM, SURREY.**—More rooms are urgently needed, and money to instal gas, both for lighting and cooking. M. Yolland, Hon. Treasurer.

**MATERNITY CHARITY AND DISTRICT NURSES' HOME, PLAISTOW, E.**—This Charity was founded in the year 1889 to nurse the sick and poor in their own homes in the districts of West Ham, East Ham, Victoria Docks, etc. The work has grown year by year, and now consists of midwifery, general district nursing, and maternity and child-welfare work. It is also the largest and perhaps the best-known training-school in the United Kingdom for this work. The following is a short statement of the work done in 1920 : Maternity cases attended, 7,744; visits, 130,080; general district cases, 2,968; visits, 109,133; maternity and child welfare (mothers and children), 18,222; visits, 63,115.

**METROPOLITAN HOSPITAL, KINGSLAND ROAD, E. 8.**—This institution is situated in one of the poorest districts in London, and is in urgent need of help. £30,000 required annually; hospital mortgage, £10,000; debt, £6,000; income from investments, less than £650; nurses' quarters inadequate and unsatisfactory; new kitchens and stores required.

**THE MIDDLESEX HOSPITAL.**—This hospital treated last year 5,438 in-patients in its general wards, and 390 in its cancer charity; in the general out-patient department there were 146,888 attendances, in the electrical department 18,545, and in the cancer charity 832; 599 new patients came to the massage department. In view of these demands upon its resources and the present excessive cost of maintenance, the hospital is especially anxious to widen its circle of permanent subscribers by bringing home to all classes of the community the benefits conferred on them, not merely on patients actually treated, by its many activities. The work in its research and pathological laboratories is of the utmost value to the health of each individual, while the medical school, with its hospital clinical teaching, helps to provide the supply of medical men necessary for the practice of medicine all the world over.

**MILDMAY MEMORIAL HOSPITAL, NEWINGTON GREEN ROAD, N. 1,** is situated in a quiet and ideal spot for the sick. Difficulties in working are great, and funds are sadly needed. Repairs are necessary, for which a large sum is required, an entirely new roof being set down by an expert as an absolute necessity. Furthermore, in case of fire, there is no extra means of escape but rope ladders from the wards ; the old iron escape is dangerous to use. At the present high cost of living it is impossible to pay for such things out of ordinary income; the hospital appeals to the public for help to supply these great needs.

**MILLER GENERAL HOSPITAL FOR SOUTH-EAST LONDON, GREENWICH ROAD, S.E. 10.**—The south-east district of London has been very sadly overlooked in the past from the point of view of hospital accommodation. Continued efforts are being made at this hospital to help to make good the deficiency. Ten years ago there were only twenty-five beds; to-day there are 100, the increase from seventy-six to that number being brought about in September last by turning the Board-room, matron's office, and another room, all adjoining, into a ward. The cost of the hospital ten years ago was only £4,500; last year it was £26,278; but the assured income from investments is only £985.

**NATIONAL HOSPITAL FOR THE PARALYSED AND EPILEPTIC, QUEEN SQUARE, W.C.**—The financial position last year was so acute that 170 beds in the main hospital had to be closed. Fortunately, timely assistance enabled the work to be started in its entirety, with a large out-patient department, the convalescent home at East Finchley, and the separate hospitals for discharged Service men, maintained by the Ministry of Pensions, at Clapham Park and Bloomsbury. Every contribution, however small, is of the utmost importance.

**NELSON HOSPITAL, MERTON** (formerly the S. Wimbledon and Merton Cottage Hospital).—This hospital was founded in 1900, in an old house in Merton Road, Wimbledon. In 1905 advantage was taken of the Trafalgar Centenary celebrations to start a fund for providing an up-to-date hospital on a central site, and to associate with it the name of Nelson, who lived at Merton for three years and intended to spend the remainder of his life there after retirement from active service. Thanks to generous support, the present hospital was erected and opened, free of debt, in 1911. Funds for maintenance are greatly needed.

**PADDINGTON GREEN CHILDREN'S HOSPITAL.**—The hospital has forty-six beds and a convalescent home at Slough with twenty-four beds. Ordinary receipts, £6,384; ordinary expenditure, £10,967. Nine hundred and thirty-seven in-patients were admitted during 1919, and 12,064 out-patients (new cases); 42,527 out-patient attendances were recorded. Total debt to-day, £6,700.

**PHILLIPS MEMORIAL HOMŒOPATHIC HOSPITAL AND DISPENSARY, BROMLEY, KENT.**—The needs of this institution include table linen of all kinds, cotton-wool dressings, and bandages. The present difficulty is a dwindling of annual subscriptions and the probability of an overdraft.

**POPLAR HOSPITAL FOR ACCIDENTS, EAST INDIA DOCK ROAD, POPLAR, E. 14.**—The Poplar Hospital for Accidents has always paid its way. The Committee strives for the strictest economy compatible with efficiency. Some very necessary rebuilding, which has been postponed since 1914, is now essential. For this purpose for some years there has been a "suspense" account, to which has been placed the balances of our income over expenditure year by year, and the hospital is now in a position to commence the work, but will require at least £15,000 more to complete the proposed scheme.

**QUEEN CHARLOTTE'S LYING-IN HOSPITAL, MARYLEBONE ROAD, N.W. 1.**—The demands for the services of the hospital increase year by year. Every year 2,000 patients are admitted to the wards, and 3,000 others are attended and nursed in their own homes. In the ante-natal and child welfare department over 5,000 patients are attended annually. The overdraft at the bank is now over £8,000. The Committee are anxious to proceed with the extension which was about to be commenced at the outbreak of war, but they are unable at present to do so without a large sum of money. A special appeal has been launched for £110,000, and up to the present £16,000 has been realised.

**QUEEN'S HOSPITAL FOR CHILDREN, HACKNEY ROAD, BETHNAL GREEN, LONDON, E. 2.**—This hospital deals with very large numbers of children of all classes that are unable to pay for suitable treatment. It is a casualty centre for an extensive area and always open. Together with its branch at Bexhill (the "Little Folks' Home") it disposes of 170 beds. but could easily use three times that number. The expenditure has risen from £16,500 in 1914 to about £35,700 at the present time, and immediate assistance is most urgently needed.

**ROYAL DENTAL HOSPITAL OF LONDON, 32 LEICESTER SQUARE, W.C. 2.**—Bank overdraft, £30; bills for payment, £879; mortgage, £21,826. There is a half-yearly instalment of £1,150, principal and interest, due to the Prudential Assurance Co. on July 1, 1921, towards which there is no money, and it is difficult to discover a means of meeting it.

**ROYAL FREE HOSPITAL, GRAY'S INN ROAD, W.C. 1.**—The financial position of this hospital is becoming critical. The expenditure during 1920 exceeded the income by no less than £19,490. A limited number of beds have been provided upon a contributory basis, but this cannot materially reduce the very heavy responsibilities of the Board of Management. To meet the immediate requirements of the hospital £40,000 is needed, and additional income from annual subscriptions is earnestly sought.

**ROYAL EYE HOSPITAL, ST. GEORGE'S CIRCUS. S.E.**—791 patients treated in the wards during 1920, 22,229 new out-patients, 4,256 casualties. Income five months 1921, £2,471; expenditure £3,655. The only ophthalmic hospital in South London, drawing patients from some of the poorest and most thickly populated districts of the Metropolis. Funds are urgently needed to prevent closing of wards.

**ROYAL MINERAL WATER HOSPITAL, BATH.**—The demands for admission are very great. The cost of maintenance and treatment is so high that unless the majority of the patients contribute wards will have to be closed shortly. A scheme for the development of research into rheumatism, arthritis, and other diseases treated at Bath is being developed by the committee.

**ROYAL NATIONAL HOSPITAL FOR CONSUMPTION, VENTNOR.**—During each of the past four years there has been (excluding legacies) a deficiency of income to meet the ordinary expenditure, amounting in the aggregate to over £4,800; whilst for the current year it is estimated that there will be a further deficit of some £5,000 or more, due entirely to the enormous increase in the cost of all necessities. The number of patients benefited by the hospital last year was 790, and of this number nearly 50 per cent. were received from London and its neighbourhood.

**ROYAL NATIONAL ORTHOPÆDIC HOSPITAL, 234 GREAT PORTLAND STREET, LONDON, W. 1.**—This hospital is making an appeal for £200,000 to provide for the extension of the present out-patient department, to establish twenty beds for paying patients, and to prepare and equip a house at the top of Brockley Hill, Stanmore, which has recently been acquired as a country branch. The out-patient attendances have increased since the present building was erected, from 11,000 to 82,000 a year, and there is an in-patient waiting-list of well over a thousand. Mr. Arthur Morley is the Secretary.

**ROYAL NATIONAL SANATORIUM FOR CONSUMPTION AND DISEASES OF THE CHEST, BOURNEMOUTH.**—"I really cannot speak too highly of the treatment I received whilst at the Sanatorium. It is nearly three years since I was a patient, and I have never had any trace of the complaint since." "My sincere thanks to doctor, matron, and staff, for the kindness and attention and the great benefit derived during my stay at the Sanatorium." "I desire to thank the doctor and nursing staff for their kindness, which has made my stay a very happy one, and for the splendid treatment which has resulted in the great improvement in my health." These and hundreds of other similar expressions of appreciation have been received from patients who have derived benefit from the treatment provided by the Royal National Sanatorium.

**ROYAL SEA-BATHING HOSPITAL, MARGATE.**—Offices: 13 Charing Cross, London, S.W. 1. The two greatest needs of this hospital at the present time are: (*a*) More trained nurses and probationers, beds being unoccupied on account of shortage of staff; (*b*) A nurses' home—and funds to provide it.

**ROYAL WATERLOO HOSPITAL FOR CHILDREN AND WOMEN, WATERLOO ROAD, S.E. 1.**—The amount at present on loan at the bank is upwards of £7,000. The greatest anxiety has been experienced in meeting the current expenses of the hospital. The new buildings, which were were commenced last year, are making rapid progress, and substantial help is needed in order to defray the monthly accounts, which exceed on an average

£3,000. The total cost is estimated at £80,000, towards which £21,000 has already been collected.

**ST. ANDREW'S HOSPITAL, DOLLIS HILL, LONDON, N.W. 2.**—Help is needed for: (1) The maintenance of the hospital; (2) the paying off of the deficit of £760 8s. 8d. on the account for 1920; (3) the extinction during 1921 of the building debt of £1,162 17s. 4d.; (4) the completion of the furnishing and equipment of the hospital in respect of the electric department and mortuary; (5) the endowment of the hospital; and (6) the completion of the building.

**ST. ANDREW'S HOSPITAL, CLEWER, WINDSOR.**—The hospital is in great need of help at the present time and cannot meet the monthly bills, in spite of strict economy, being considerably overdrawn at the bank. This year we have to meet the expense of putting in electric light and to replace the boiler for heating the hospital, which was completely worn out. A gift of £500 would meet these extra expenses.

**ST. BARTHOLOMEW'S HOSPITAL, SMITHFIELD, E.C. 1.**—"Bart's" is the Empire's oldest hospital, and the only general hospital in the City of London. £180,000 is required annually for maintenance, about half of which is derived from invested funds. Contributions are urgently asked for.

**ST. COLUMBA'S HOSPITAL, OR HOME OF PEACE, 98 AVENUE ROAD, SWISS COTTAGE.**—The hospital is not intended for chronic invalids, but for dying patients, for whom no provision is made in the general hospitals. Since the war the expenses have nearly doubled, and the contributions have not kept pace. There is a debt of nearly £2,000, in spite of the fact of an appeal having been issued last year. The forecast shows that unless any unforeseen donations are received this debt will be considerably increased by December 31 next.

**ST. GEORGE'S HOSPITAL, S.W. 1.**—This Hospital, which was established 188 years ago, is still carrying on the work then commenced for the sick poor. How enormously this has increased may be seen from the fact that in 1734 (the first completed year) 371 in-patients and 258 out-patients were treated, whereas in 1920 4,929 in-patients and 37,871 out-patients received treatment. The ordinary expenditure exceeded the ordinary income in 1920 by £35,754, and as there seems every likelihood of a similar deficit this year, the kind assistance of all who may have the welfare of this hospital at heart is earnestly desired.

**ST. JOHN'S CONVALESCENT HOME FOR CHILDREN, WALPOLE ROAD, BRIGHTON.**—St. John's Home for Convalescent Children, which has carried on for upwards of fifty years such valuable recuperative work among the children of East London and other large towns, is going through an anxious period of need of funds and is shadowed by the dread possibility of having to close down, though it is earnestly hoped that this latter contingency may, through careful management and a fair response to public appeal, be avoided.

**ST. JOHN'S HOSPITAL FOR DISEASES OF THE SKIN, LEICESTER SQUARE, W.C. 2.**—Funds are urgently needed to provide new accommodation for the nurses and to reopen the children's ward. £800 is in hand towards the £2,000 required. This hospital was a pioneer of the patients' payment system, and was in consequence looked upon as a pariah outside the pale of respectable hospitals. In consequence of this it was, over a period of many years, mulcted to the extent of thousands of pounds for obtaining funds in a manner now gladly adopted by nearly every hospital in the kingdom.

**ST. JOHN'S HOSPITAL, MORDEN HILL, LEWISHAM.**—This hospital has practically no endowment, and depends for its existence on donations and subscriptions. New nurses' quarters and a children's ward are absolutely necessary. The hospital is free, and open to all, and no restriction as to creed or nationality of patients exists.

**ST. LUKE'S HOSPITAL FOR ADVANCED CASES (A HOME FOR THE DYING), 14 PEMBRIDGE SQUARE, BAYSWATER, W. 2.**—For cases of mortal illness in its latest phase. To provide skilled nursing and medical attendance, with the comforts of home. Admission free, irrespective of religious denomination. In addition to subscriptions and donations, which are required for the maintenance of the hospital, funds are urgently needed for the building fund, which at present amounts to only £11,309.

**ST. MARK'S HOSPITAL, CITY ROAD.**—The sum of £7,000 is required to pay off a loan and to meet the ordinary annual expenditure. Funds are also needed for the building of additional nurses' bedrooms, which will cost £2,500. All contributions will be most thankfully received by Mr. Robert Holland-Martin, C.B., the Treasurer.

**ST. MARY'S CONVALESCENT HOME, BIRCHINGTON-ON-SEA.**—This home is in need of help, especially at this moment, as it has been found necessary to renew the boiler and the whole of the hot-water heating system throughout. A donation would be most thankfully received by Hon. Lady Fremantle, 10 Draycott Place, S.W. 3.

**ST. MARY'S CONVALESCENT HOME, BROADSTAIRS.**—Through the courtesy of the Editor of THE HOSPITAL, and in the interest of over 1,000 invalid children yearly, we most earnestly ask help for this Convalescent Home, situated in one of the most important health resorts of England. After the exceptionally difficult year of 1920, we find the work overshadowed with a debt of £946 15s. 7d., a very serious burden to those who have charge of the Home and its little inmates.

**ST. MICHAEL'S CONVALESCENT HOME, WESTGATE-ON-SEA,** is the convalescent home of the London Diocesan Deaconess Institution (St. Andrew's Deaconess Community, Tavistock Crescent). The Home is open to persons of any religious denomination, and is intended for necessitous men and women of the middle and artisan classes. There are thirty-six beds. The great need of the Home now is a larger circle of friends and subscribers.

**ST. PETER'S HOSPITAL, HENRIETTA STREET, COVENT GARDEN, W.C. 2.**—Continued help and new annual subscriptions are urgently needed. This year it will be absolutely necessary to paint the outside and do certain internal painting and repairs to the building, the cost of which will approach £1,000, and an earnest appeal is therefore made for funds to meet this special expense.

(*Continued on p.* 20.)

### HOSPITALS AND THEIR SPECIAL NEEDS.

(*Continued from p.* 18.)

**SANTA CLAUS HOME, CHOLMELEY PARK, HIGHGATE, LONDON, N.**—This Surgical Nursing Home for poor children is in urgent need of funds, owing to the greatly increased cost of maintenance. Children with hip and spinal diseases, and other surgical cases needing skilled nursing, long rest, and care, are taken. During the thirty years the Home has been open the results have been most satisfactory, the majority of the children returning to their own homes fit for school or work. Donations to be sent to Miss Charles at the Home.

**SOUTH-EASTERN HOSPITAL FOR CHILDREN, SYDENHAM.**—At the present time this hospital has borrowed from the bankers £1,300 in order to carry on. It is necessary to rebuild the out-patients' department, which is totally inadequate for the treatment of 12,000 patients annually; plans have been prepared, and the cost will be some £10,000. An effort to raise a large part of this sum will shortly be made.

**SOUTH LONDON HOSPITAL FOR WOMEN AND CHILDREN, SOUTH SIDE, CLAPHAM COMMON, S.W.**—The need for a substantial increase in voluntary contributions is particularly urgent owing to the deficit in the income for the past year. There is also a debt of £500 on building and equipment account. A considerable sum has been expended this year on repainting and other necessary improvements. All patients are expected to contribute according to their means towards the cost of their maintenance whilst under treatment, but it is impossible to secure from this source a sufficient amount to meet the deficit in income.

**TEDDINGTON AND HAMPTON WICK COTTAGE HOSPITAL.**—Founded in 1875 and has twenty-four beds. Except for the services of the matron and the staff, all work done for the hospital, including that of the medical staff, is voluntary. The hospital building is an old one, and is inadequate for the present requirements of the district and modern treatment. The trustees have acquired a suitable site for a new hospital, for which funds are urgently needed.

**UNIVERSITY COLLEGE HOSPITAL, GOWER STREET, W.C.**—This hospital has not previously made a general appeal to the public, but this step is now found necessary for the following reasons: (1) The hospital has gradually got back to the state of normal work, and those beds which were given over to nursing war casualties are now available for general purposes, and the cost of general upkeep is therefore correspondingly increased. (2) Cost of maintenance has risen in every branch, and the deficit on working in 1920 was £10,000. (3) The acceptance of the Rockefeller Gift has provided a sum of £400,000 for a building scheme which will increase the capacity of the hospital by some 180 beds. This will mean the raising of an additional income for maintenance of a minimum of £20,000. The princely Rockefeller gift for the purposes of medical education conveys a silent message to the British public to contribute its share to a great imperial scheme in the cause of humanity, and the committee feel sure that this message from a sister nation will meet with a prompt and generous response. Mr. W. D. Nixon is the Appeal Officer.

**VICTORIA HOSPITAL, CHELSEA.**—The ordinary income for 1920 fell short of the total expenditure by £8,670 14s. 6d., the deficit at the end of the year being reduced to £3,603 owing to legacies (£3,552) and unexpected emergency grants from King Edward's Hospital Fund and the National Relief Fund. These receipts being non-recurrent, the outlook for 1921 is exceedingly grave. The deficit on the Convalescent Home Account amounted to £1,483. Various proposed improvements, although urgently needed, have been unavoidably postponed through lack of funds.

**WESTMINSTER HOSPITAL.**—The ordinary income in 1920 fell short of the expenditure by £24,456, and but for the special grants of £9,500 and £11,500 made by the King Edward's Hospital Fund for London and the National Relief Fund respectively, the hospital would be in serious financial difficulty. The excess of expenditure over income for the first quarter of this year was £5,000, and the committee earnestly appeals for donations and subscriptions.

